# Scientific Items

**Published:** 1886-02-20

**Authors:** 


### SCIENTIFIC ITEMS.

**To Glue Leather to Iron.**—(Scientific American) There is a constant inquiry as to the best plan for fastening leather to iron, and there are many recipes for doing it. But probably the simplest mode, and one that will answer in a majority of cases, is the following: To glue leather to iron, paint the iron with some kind of lead color, say white lead and lamp black. When dry, cover with a cement made as follows: Take best glue, soak it in cold water till soft, then dissolve it in vinegar with a moderate heat, then add one-third of the bulk of white pine turpentine, thoroughly mix, and by means of the vinegar make it of the proper consistency to be spread with a brush, and apply it while hot; draw the leather on quickly, and press it tightly in place. If a pulley, draw the leather around tightly, lap, and clamp.

**Water is Fattening.**—(Scientific American) It has been observed that water is fattening, that those who drink large quantities of water have a tendency to rotundity. That there is considable truth in this observation the *Medical and Surgical Reporter* fully substantiates. That excessive imbibition of very cold (iced) water (especially when one is very warm) is not to be commended, yet we have reason to believe that *unlimited* use of *pure spring water*, at its natural temperatuie, is not only very conducive to health, but has an actual tendency to favor a fullness and roundness of body. Whether this is the result of a better action on the part of the digestive, assimilative, and depurative functions, owing to the interal cleanliness or flushing of the human sewers produced by large quantities of water, or whether water has some specific action in producing this fullness, we do not know, neither does it signify, since observation confirms as a fact that the free use of water does have this effect.

**Insusceptibility of a Dog to the Action of Hydrocyanic Acid.**—(National Druggist) Recently it was my unpleasant duty to destroy the lives of two favorite dogs, and hydrocyanic acid was the agent chosen.

I gave to one dog, an old retriever, 4 drams of the acid, and death took place in about five minutes.

To the other was given the remaining 4 drams from the same bottle without the slightest apparent effect.

I had, fortunately, provided myself with two one-ounce bottles, and at the end of ten minutes I administered the remaining ounce of acid. After a few minutes the respiration became spasmodic, and violent convulsions set in, a film covering the eye, which was greatly contracted, and I thought death close at hand. But after some minutes of distressing pain, followed by coma, the dog showed unmistakeable signs of recovery, and at the end of twenty minutes the action of the poison seemed to have ceased. My assistant then killed the dog by a process more summary than scientific.

**The Moon and Us**—(Scientific American) The first of a series of scientific lectures, to be delivered before the Science Matinee Club, at the Hotel Brunswick, New York, was that by Prof. Young, of Princeton, on the moon, which was illustrated by the stereopticon. The lecturer spoke of our satellite as the petrified daughter of the earth, since it is destitute of life, air and water.

The moon always has been a favorite subject of study among astronomers, on account of its proximity and because it is the only heavenly body, with the exception of the sun, that exercises an appreciable influence upon our planet. The lunar temperature is one of violent extremes. In the dark spots, under the shadow of the lunar Alps, it is calculated to be about 200° below zero; while in the localities exposed to the sunlight, the temperature of boiling water is supposed to prevail. Beyond her influence upon the tides, the moon has little power in earthly affairs, in spite of the popular belief in her distributing action upon the human brain or her assistance in the germination of the sown grain. Were she annihilated, the temperature of New York, Professor Young said, would be reduced 1°. In her present orbit, however, she has absolutely no influence upon weather. In conclusion, the lecturer begged artists not to paint their crescent moons upside down, as Hogarth has done in one of his pictures.

**The** Chemists and Druggist says that Sir John Lubbock's report on his ants, wasps, and bees, at the British Pharmaceutical Conference, was immensely popular. One of the most interesting points connected with the ants was the manner in which they recognized their friends. Not only would the ants in any nest, however large, distinguish between their own companions and those belonging to the same species, but this happened even after a separation of more than a year. As regarded their longevity, he had two which he kept ever since 1874. They were then full grown, and must, therefore, be twelve years old. They were both queens, and continued to lay eggs, showing no sign of age, excepting, perhaps, that they were a little stiff in the joints.—*National Druggist.*

**Naturalists** say that the feet of the common working-bee exhibit the combination of a basket, a brush and a pair of pincers. The brush, the hairs of which are arranged in symmetrical rows, are only to be seen with the microscope. With this brush of fairy delicacy the bee brushes its velvet robe to remove the pollen dust with which it becomes loaded while sucking up the nectar. Another article, hollowed like a spoon, receives all the gleanings which the insect carries to the hive. Finally, by opening them, one upon another, by means of a hinge, these two pieces become a pair of pincers, which render important service in the construction of the combs.—*The Medical Advocate.*

